# Usefulness of Aquaporin 1 as a Prognostic Marker in a Prospective Cohort of Malignant Mesotheliomas

**DOI:** 10.3390/ijms17071041

**Published:** 2016-06-30

**Authors:** Jack Driml, Emily Pulford, David Moffat, Christos Karapetis, Steven Kao, Kim Griggs, Douglas Warrington Henderson, Sonja Klebe

**Affiliations:** 1 Department of Anatomical Pathology, Flinders University of South Australia, Bedford Park, South Australia 5042, Australia; jack.driml@sa.gov.au (J.D.); pulf0010@flinders.edu.au (E.P.); kim.griggs2@sa.gov.au (K.G.); 2 Department of Surgical Pathology, SA Pathology at Flinders Medical Centre, Bedford Park, South Australia 5042, Australia; david.moffat@sa.gov.au (D.M.); dhenderson@internode.on.net (D.W.H.); 3 Medical Oncology, Flinders Medical Centre, Bedford Park, South Australia 5042, Australia; chris.karapetis@sa.gov.au; 4 Asbestos Diseases Research Institute, Bernie Banton Centre, Concord Repatriation General Hospital, Concord 2139 Sydney, Australia; Steven.Kao@lh.org.au

**Keywords:** malignant mesothelioma, pleura, peritoneum, Aquaporin 1, asbestos, prognosis

## Abstract

(1) Background: Malignant mesothelioma (MM) is an aggressive tumour of the serosal membranes, associated with exposure to asbestos. Survival is generally poor, but prognostication for individual patients is difficult. We recently described Aquaporin 1 (AQP1) as independent prognostic factor in two separate retrospective cohorts of MM patients. Here we assess the usefulness of AQP1 prospectively, and determine the inter-observer agreement in assessing AQP1 scores; (2) Methods: A total of 104 consecutive cases of MM were included. Sufficient tissue for immunohistochemistry was available for 100 cases, and these cases were labelled for AQP1. Labelling was assessed by two pathologists. Complete clinical information and follow up was available for 91 cases; (3) Results: Labelling of ≥50% of tumour cells for AQP indicated improved prognosis in a univariate model (median survival 13 versus 8 months, *p* = 0.008), but the significance was decreased in a multivariate analysis. Scoring for AQP1 was robust, with an inter-observer kappa value of 0.722, indicating substantial agreement between observers; (4) Conclusion: AQP1 is a useful prognostic marker that can be easily incorporated in existing diagnostic immunohistochemical panels and which can be reliably interpreted by different pathologists.

## 1. Introduction

Malignant Mesothelioma (MM) is an aggressive malignancy of the serosal membranes lining the pleural, peritoneal and pericardial cavities. The current incidence of MM is attributable overwhelmingly to asbestos exposure and is predicted to remain stable or even increase into the near future, due to the continued presence of asbestos in buildings and long latency post-exposure (up to several decades). Prognosis is poor, due to limited response to standard treatment strategies [1], and prognosis for individual patients is difficult to predict. This signifies the need for reliable prognostic markers, which can be easily included in the clinical work-up of the patient [2].

Established prognostic indicators in MM include the histological subtype, and sex and age at diagnosis. More recently, neutrophil to lymphocyte ratios, serum inflammatory markers, as well as serum, tissue and pleural effusion Vascular endothelial growth factor (VEGF) levels and and BRCA1-Associated Protein 1 (BAP1) mutation BAP1 status have been related to outcomes among MM patients [2,3,4,5,6]. We recently described Aquaporin 1 (AQP1) as an independent prognostic factor in multivariate analysis in two retrospective independent cohorts of MM that were treated either conservatively or by aggressive surgery [7].

Aquaporins (AQPs) are a family of transmembrane water channel proteins with roles in water transport, cell proliferation, and pain perception. Expressed on tumour cells and vascular endothelial cells, AQPs are investigated for their use in cancer prognosis, and they also represent a potential target for cancer therapy [8,9,10,11]. Aquaporin 1 is over-expressed in some MMs [12]. Related to its function as a water channel that mediates the fluid balance of endothelial and epithelioid cell types, a role in pleural effusion fluid accumulation has been proposed [13,14].

## 2. Results

### 2.1. Immunohistochemical Analysis

Surgical blocks of 104 consecutive cases of MM were received, and 100 cases for which sufficient material was available were labelled prospectively by immunohistochemistry for AQP1. Sufficient tissue was defined as 4 mm^2^ of tumour tissue, based on our previous findings in tissue mircoarrays [7]. The number of cells assessed for labelling varied, depending on the size of the sample and the histological subtype of the tumour. Clinical information and follow up was available for 91 cases (see Table 1 for baseline characteristics).

### 2.2. AQP1 as a Prognostic Indicator

**Figure 1:**
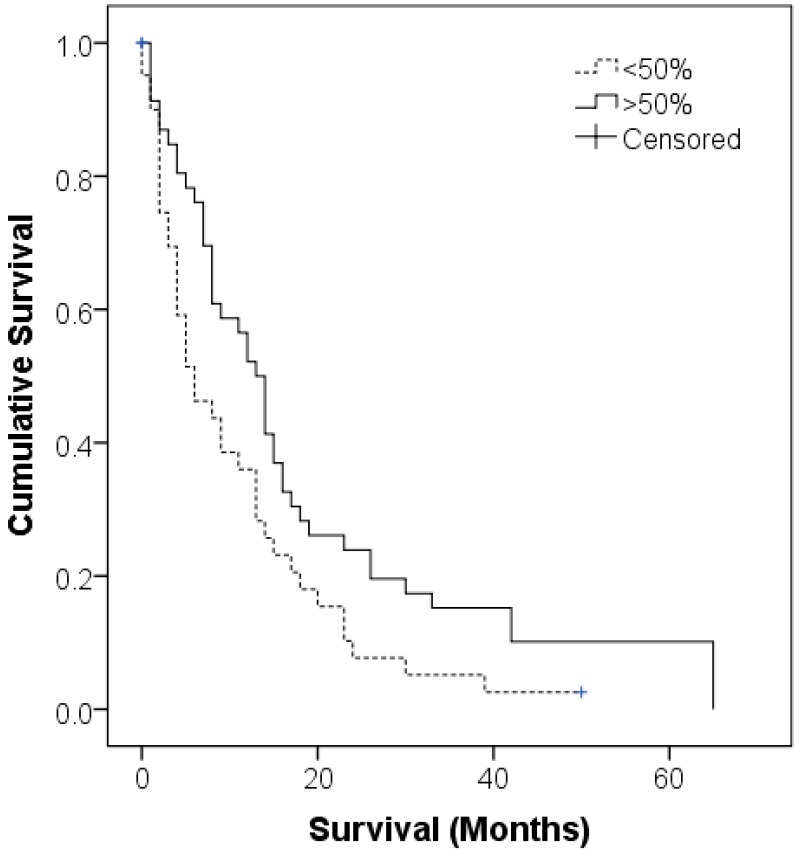
Kaplan Meier curve of Aquaporin 1 (AQP1) expression in this cohort of 91 malignant mesothelioma (MM) patients. The median overall survival was 13 months (95% CI 7.9–18) for patients with ≥50% AQP1 expression, *n* = 4, versus a median overall survival of 8 months (95% CI 4–12) for patients with <50% AQP1 expression (*n* = 7).

The prognostic value of patient AQP1 status was confirmed in the Cox regression univariate analysis (*p* = 0.008), however this was not reflected in the multivariate model (*p* = 0.233) within this cohort (Table 2). Median patient survivals for patients with <50% (*n* = 47) and ≥50% (*n* = 44) tumour cells expressing AQP1 were 13 (95% CI 7.9–18) and 8 (95% CI 4–12) months respectively, using the Kaplan Meier method (Figure 1).

There was no significant difference in the distribution of AQP1 scores between pleural and peritoneal MMs, but only six peritoneal MMs were included (Table 3).

In this cohort, 70% of cases of epithelioid MM had ≥50% AQP1 scores whereas only 7% of sarcomatoid and 8% of biphasic MMs had AQP1 scores of ≥50% (Table 4).

### 2.3. Established Prognostic Indicators

Our cohort included six peritoneal MMs, all epithelioid in type and which did not show significantly different survivals from our pleural MMs, with median survivals being 9 months for both pleural and peritoneal MMs (but mean survivals were 13.6 and 20.7 months, respectively).

Previously established and recognized prognostic factors in MM, including histological subtype and patient age were significantly associated with overall survival in this cohort [2]. As expected, the sarcomatoid and biphasic subtypes were significantly associated with poorer survivals. Median survival for epithelioid MM was 13.5 months versus 4.6 months for biphasic and 2 months for sarcomatoid subtypes (Table 5, *p* = 0.003 and *p* = 0.022 respectively compared to the epithelioid subtype in univariate analysis).

The sarcomatoid subtype was maintained as a significant prognostic indicator in the multivariate model (*p* = 0.014). Age equal or greater than the median of 71 years was also associated with poorer survival in univariate and multivariate analysis (*p* = 0.014 and *p* = 0.007 respectively). Although male patients often exhibit poorer prognosis than females [15], this was not applicable in our cohort. Likewise, the different treatment strategies including surgery, chemotherapy and radiotherapy were not significantly associated with overall survival. Some patients underwent two treatment modalities, e.g., chemotherapy and radiotherapy.

### 2.4. Reproducibility of AQP Scoring in Histological Sections

Labelling for IHC, defined as membrane labelling, was assessed by two pathologists (SK1 and DM) as less than or equal to, or more than, 50% of tumour cells. Agreement between pathologists' assessment was measured using the kappa statistic, which in this case was 0.772, indicative of substantial inter-observer agreement [16] and highlighting that scoring for AQP1 is robust.

## 3. Discussion

AQP1 is a membrane-bound water channel protein which has roles in cell proliferation and migration, as well as fluid homeostasis [17]. We have previously demonstrated AQP1 as a significant prognostic indicator in two retrospective cohorts of MM [7]. In this current prospective cohort, AQP1 was maintained as a significant indicator of prognosis in univariate analysis, but significance was lost in multivariate analysis, with scores being related to histological subtype. This confirms our previous findings, where we found a statistically significant difference in levels of AQP1 expression between different histological subtypes of MM (*p* < 0.001) [7]. Because AQP1 is expressed in normal mesothelium, and the level of differentiation in MM is thought to decrease from epithelioid to biphasic to sarcomatoid subtypes, it is conceivable that loss of AQP1 expression is an indication of further tumour de-differentiation.

Normal mesothelial cells express AQP1 at the apical aspect of the cells, whereas MMs show loss of polarity of AQP1 expression, or a complete loss of AQP1 labelling [7]. The situation in MM, where higher levels of AQP1 are related to better prognosis is unlike that in other tumours, where increased levels of AQP1 are associated with poorer prognosis, including breast cancer, melanoma, urothelial carcinoma and pharyngeal squamous cell carcinomas [18,19,20,21,22]. Interestingly, in some of these tumours AQP1, a membrane bound protein, was found within the cytoplasm [21]. It may be that the retained expression of AQP1 in MM relates to a degree of preserved differentiation, while complete loss (as in most of the sarcomatoid MMs) indicates a lesser level of mesothelial differentiation and enhanced aggressiveness. However it is worth noting that, like other tumours, inhibition of AQP1 by either synthetic blocker or siRNA resulted in decreased MM cell motility and invasiveness in standard culture and spheres [14,23]. However, AQP1 is also expressed by vascular endothelial cells, and blockade of AQP1 disrupts vessel formation in a number of models [24,25,26].

Although male MM patients often exhibit poorer prognosis than females [15], this was not so in our cohort. This may potentially due to gender bias, reflecting the differential incidence of MM within populations. Likewise, treatment strategies including surgery, chemotherapy and radiotherapy were not significantly associated with overall survival, although the majority of this cohort underwent conservative treatment, with less radical methods, and for which a lesser impact on survival would be expected.

## 4. Materials and Methods

### 4.1. Patient Recruitment

This prospective cohort consists of 104 MM patients who were diagnosed or reviewed at the Flinders Medical Centre from 2010 to 2013. For 91 of these patients, sufficient clinical follow up was available. Histological subtype was assessed according to the World Health Organization criteria [27]. Surgical blocks were available through SA Pathology. Patients were included based on a histological diagnosis of MM, availability of tissue in paraffin blocks, and availability of clinical follow-up information. Patients for whom no clinical follow up was available, or where insufficient material was available in the block were excluded. This work was approved by the Southern Adelaide Clinical Human Research Ethics Committee (approval number 381.09, 2 May 2009).

### 4.2. Immunohistochemical Analysis

Paraffin sections were cut 4 µm thick, deparaffinized and rehydrated prior to quenching with 1% H_2_O_2_. The sections were incubated with 1:6000 rabbit anti-rat AQP1 IgG (Alpha Diagnostics, Cat #AQP11-A San Antonio, TX, USA) overnight at 4 °C. Detection was performed using Novolink Max Polymer Detection System (Leica Biosystems, North Ryde, Australia) and Liquid DAB+ Substrate Chromogen System (Dako Australia Pty Ltd., North Ryde, Australia). For the quantitative evaluation, the percentage of cells labelled by the antibody was assessed by two qualified anatomical pathologists (SK1 and DM) independently, irrespective of the intensity. Only membrane labelling was considered specific and was confirmed from 10 high-power (40×) fields. However, the percentage of cells that labelled was estimated from scanning. The whole slide was scanned at 20× power, since labelling can be variable in some tumours, and therefore scanning of the whole slides was considered more accurate than random fields. Only labelling in tumour cells was scored, and labelling in vessels (vascular endothelial cells are known to express AQP1) was not taken into account. Neither investigator was aware of the survival data when scoring was assessed. This resulted in a percentage score that ranged from 0% to 100%. For all cases, one representative slide was assessed for labelling. Most cases were diagnostic biopsies, and only one block was available for the majority.

### 4.3. Statistics

Statistical analysis was performed on IBM SPSS statistics version 2.22 (IBM Australia, St Leonards, Australia). Kappa (κ) scores were used to evaluate agreement between percentage assessments by pathologists. Survival was quantified as months between diagnosis and death of the patient, and was analysed using Kaplan-Meier curves, and Cox Regression univariate and multivariate multivariate analysis. Other established prognostic factors in MM, including age, sex and histological subtype were entered into the multivariate model. A difference was accepted as significant if *p* < 0.05.

## 5. Conclusions

In summary, we confirm that AQP1 is a useful prognostic marker in MM, which is reliably scored with substantial inter-observer agreement. There was significantly improved survival fpr patients with ≥50% AQP1 expression in univariate analysis, whereas AQP1 expression was inseparable from the sarcomatoid type in multivariate analysis.

## Figures and Tables

**Table 1:** Patient Baseline Characteristics (91 patients).

Variable		Count (%)
**Age in Years, Median (Range)**		71 (40–91)
**Sex**	Male	71 (78)
	Female	20 (22)
**Type**	Pleural	85 (93)
	Peritoneal	6 (7)
**Subtype**	Epithelioid	66 (73)
	Biphasic	11 (12)
	Sarcomatoid	14 (15)
**Treatment (Some patients had 2 treatment modalities)**	Radical surgery (EPP)	1 (1)
	Chemotherapy	17 (19)
	Radiotherapy	6 (7)
	Conservative	71 (76)
**AQP1 Score**	<50%	47 (52)
	≥50%	44 (48)

Baseline characteristics for 91 patients with malignant mesothelioma and clinical follow up.

**Table 2:** Cox Proportional Hazards Model for Survival Analysis.

Variable		Univariate		Multivariate	
		HR	*p*	HR	*p*
**Age**	<71	1.0		1.0	0.007
	>71	1.754		1.893	
**Sex**	Female	1.0		1.0	0.813
	Male	1.011	0.968	1.061	
**Subtype**	Epithelioid	1.0		1.0	
	Sarcomatoid	2.757	0.003	2.633	0.014
	Biphasic	2.048	0.022	1.752	0.099
**AQP1 Status**	<50%	1.830	0.008	1.367	0.233
	>50%	1.0		1.0	

HR, Hazard ratio; CI, Confidence interval. Included are 91 patients with complete follow-up information.

**Table 3:** Distribution of AQP1 scores in pleural and peritoneal MM was not significantly different. Included in this table are all 100 cases for which sufficient tissue for immunohistochemistry was present in the blocks, irrespective of clinical follow up.

AQP1 Score	Pleural	Peritoneal
<50%	43	4
≥50%	51	2

**Table 4:** Distribution of AQP1 scores between the different histological subtypes of MM. Included in this table are all 100 cases for which sufficient tissue for immunohistochemistry was present in the blocks, irrespective of clinical follow up.

AQP1 Score	Epithelioid	Biphasic	Sarcomatoid
<50%	22	12	13
≥50%	50	2	1

**Table 5:** Significance of established prognostic factors in this cohort- the 91 cases with complete clinical follow up are included.

Survival Statistic	Epithelioid	Biphasic	Sarcomatoid
Mean	16.7	7.6	6.2
Median	13.5	4.5	2.0
Minimum	0	0	0
Maximum	65	24	39
